# On the Definition of Signal-To-Noise Ratio and Contrast-To-Noise Ratio for fMRI Data

**DOI:** 10.1371/journal.pone.0077089

**Published:** 2013-11-06

**Authors:** Marijke Welvaert, Yves Rosseel

**Affiliations:** Department of Data Analysis, Ghent University, Gent, Belgium; University of Minnesota, United States of America

## Abstract

Signal-to-noise ratio, the ratio between signal and noise, is a quantity that has been well established for MRI data but is still subject of ongoing debate and confusion when it comes to fMRI data. fMRI data are characterised by small activation fluctuations in a background of noise. Depending on how the signal of interest and the noise are identified, signal-to-noise ratio for fMRI data is reported by using many different definitions. Since each definition comes with a different scale, interpreting and comparing signal-to-noise ratio values for fMRI data can be a very challenging job. In this paper, we provide an overview of existing definitions. Further, the relationship with activation detection power is investigated. Reference tables and conversion formulae are provided to facilitate comparability between fMRI studies.

## Introduction

In science and engineering, the signal-to-noise ratio (SNR) is a measure that compares the level of a desired signal to the level of background noise. For data acquired through magnetic resonance imaging (MRI), this quantification is typically used to allow comparison between imaging hardware, imaging protocols and acquisition sequences. In this context, SNR is conceptualised by comparing the signal of the MRI image to the background noise of the image [1], [2]. Mathematically, the SNR is the quotient of the (mean) signal intensity measured in a region of interest (ROI) and the standard deviation of the signal intensity in a region outside the anatomy of the object being imaged (i.e. a region from which no tissue signal is obtained) or the standard deviation from the noise distribution when known (e.g. the background noise in magnitude data follows a Rician distribution [3]). By varying, for example, field of view, scan parameters, magnetic field strength and slice thickness, the SNR of MRI images can be increased because these parameters influence the background noise. On the other hand, scanning hardware has also a major influence on the SNR levels of fMRI data. For example, SNR increases almost linearly with field strength [4] and is largely effected by the receiver coils (see [5] for a review).

Translating SNR of MRI images to fMRI images is not as straightforward as it may seem. First of all, the noise in fMRI images does not correspond to the background noise of MRI images (see [6] for an excellent overview). In fMRI images, system noise effects the image as well as noise stemming from the subject (i.e. cardiac and respiratory pulsations, motion) and the task that is performed. Using time series outside the brain as noise measurement only, will not be sufficient to capture the noise data [1], [7], [8]. Secondly, since the main goal of fMRI studies is to detect small fluctuations over a period of time, image SNR might not be suitable. Therefore, temporal SNR (tSNR), in which the (mean) signal over time is taken into account, can be used to determine the SNR of fMRI time series [9].

How to define SNR for MRI and fMRI data is documented quite well in terms of its relationship with the MRI acquisition parameters. Several studies have demonstrated the dependence on these parameters and illustrated the necessary conditions to obtain higher SNR [1], [7], [10]. Also the relationship between SNR and CNR (contrast-to-noise ratio, a measure of image quality based on a contrast rather than the raw signal) has been investigated. For example, Wald investigated the improvement of BOLD CNR at higher field strength [11]. He showed that BOLD CNR is not directly affected by most acquisition parameters such as reciever coil choice, parallel imaging acceleration and voxel size, but only through the influence on the tSNR. However, in the end, one is interested in how well the experimentally induced activation can be detected. From a statistical perspective, it is not entirely clear how the SNR measurements relate to this detection power, because the small activation fluctuations (typically around 1–5%) cannot be derived from the mean signal based on a static image or time series. So for fMRI data, using the CNR of the time series instead of (t)SNR is more preferred because CNR compares a measure of the activation fluctuations to the noise [12]. In short, image SNR should be used to assess data quality of a single image, while tSNR gives information on the data quality of fMRI time series. CNR on the other hand, provides knowledge on how easy, or hard, it is to detect experimentally induced signal fluctuations.

These different conceptualisations of SNR make it hard to compare results over studies. Therefore, in this paper, we provide an overview of the most current SNR definitions for fMRI. First, we demonstrate the use of these definitions in the literature and discuss thoroughly how the definitions can be applied. Second, we show analytic results that will enable the comparison between the several definitions. Third, we also analyzed the relationship with activation detection power and present simulation results that clarifies this relation. Finally, an application on real data illustrates how the definitions can be used in practice.

## Methods

To retrieve the range of possible values of SNR and CNR, we looked at the reported values of SNR and CNR in fMRI studies. NeuroImage published in 2012 about 458 fMRI studies that presented original fMRI data. These papers were manually screened if they reported SNR/CNR values of their data. In total, 50 of these studies mentioned the role of SNR/CNR for their experiment or method, while only 18 papers also reported SNR or CNR values.

Since the determination of the SNR and CNR of real data can be a demanding job and is not standardly reported, we also looked at the SNR/CNR values that were reported in simulation studies. In simulation studies, the range of the reported SNR/CNR values was determined based on a representative sample of 119 articles describing at least one simulation study. This sample containes papers that were published between 1996 and 2012 in about 49 scientific journals. The reported values varied widely across studies and were almost exclusively labelled as SNR.

### Simulation study

A simulation study was conducted to investigate the relationship between SNR/CNR levels and the power to detect activation in a basic GLM analysis. 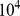 time series were simulated for three types or experimental design: (1) a block design, (2) an event-related (ER) design, and (3) a contrast between two conditions. These experimental designs serve as basic templates. More complex designs can be partially reduced to one of these three design types based on the specific research hypotheses at hand (i.e. a specific contrast or the effect of a specific predictor).

An activation signal of 200s was modelled for each design. The block design consisted of alternating task and rest blocks that lasted 20s each. For the ER design, 25 events were randomly distributed over the whole time series. For the contrast, two alternating block conditions of 20s each were modelled with a rest period of 20s after each sequence 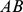 and the effect of condition 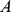 was twice as high as the effect of condition 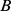. Activation time series were the result of convolving the stimulus boxcar function with the canonical HRF. The baseline value of these time series was considered fixed at 100 and we chose three levels of percent signal change, 1%, 2%, and 5% respectively. Random Gaussian noise was added to these time series and the standard deviation of the noise was allowed to vary between 0.1 and 10.

The empirical power was determined by fitting a standard GLM model to each of the simulated time series. In both the block and the ER design, the power was assessed by testing 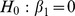. For the contrast design, 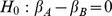 was tested. The obtained power is defined as the average number of correct detections (i.e. rejection of null hypothesis) over all time series. All simulations were carried out using the R package neuRosim [13].

### Data acquisition

As an illustration of the discussed definitions, we applied the SNR/CNR definitions in the context of experimental fMRI data and resting-state fMRI data. The experimental fMRI dataset is based on a houses-faces object recognition paradigm [14] and is freely available through the OpenfMRI project (http://openfmri.org). Forty 3.5-mm-thick sagittal images were acquired on a GE 3T scanner (General Electric, Milwaukee, WI) with TR  =  2500 ms, FOV  =  24 cm, TE  =  30 ms and flip angle  =  90. This resulted in 1452 volume scans. For the resting state data, we randomly selected one subject (AnnArbor-sub04111) from the 1000 Functional Connectomes project [15]. These data, consisting of 295 resting scans, were acquired at 3T field strength with a voxel size of 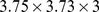, a matrix of 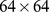 and TR of 2s.

## Results

### Reported SNR values in the literature

**Figure 1:**
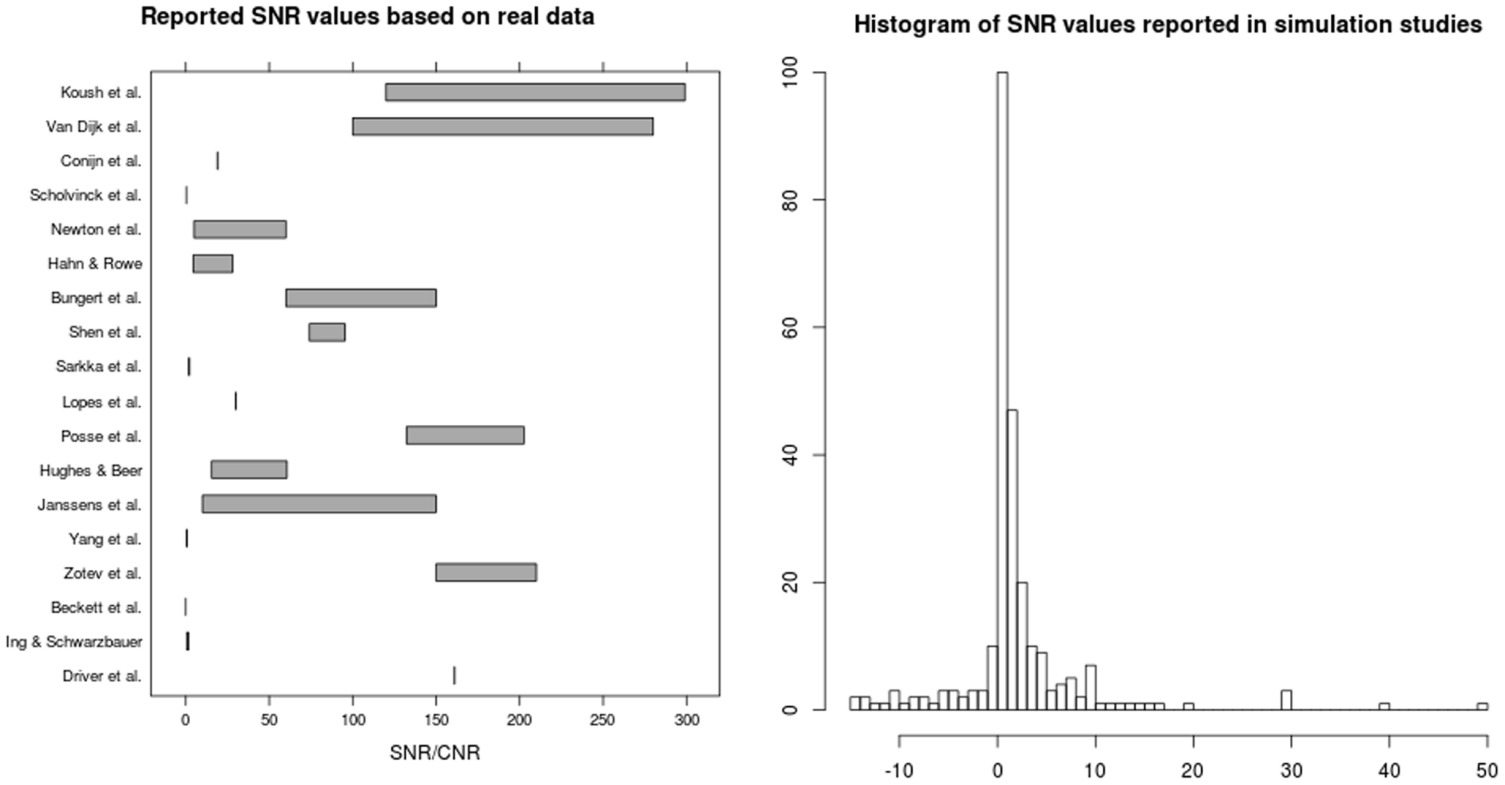
Overview of reported SNR values in real data (left panel) and simulated data (right panel).[17], [46]–[61].

Reported SNR values ranged over studies from 0.35 to 203.6 for real fMRI data[17], [46]–[61]. An overview of the values that were reported in these studies is presented in Figure 1. Many authors explicitly reported tSNR values ranging from 4.42 to 280, while in a few other cases CNR values were reported that varied from 0.5 to 1.8. Note that one study reported the possibility of a CNR value as low as 0.01, but this was specific to the imaging of orientation columns in the visual cortex and caused by a combination of bias and voxel size [16]. An interesting observation was that Hughes and Beer made an explicit distinction between SNR for active clusters and SNR for non-active clusters [17].

In the simulation studies, the reported values varied widely across studies and were almost exclusively labelled as SNR. For example, the SNR for the simulations varied from 1 to 10 in one study, while the range was 0.01 to 1 in another, and in yet other studies, we found SNR values that could be negative, for instance, ranging from −13 to 30.

Both in the experimental and simulation studies in our literature search, the reported values demonstrated a range that was much wider than can be explained by natural variation only. There is only one reason that could account for the found variation, namely, the use of different definitions to calculate SNR or CNR. Indeed, several definitions can be found in the literature, especially for CNR. All these CNR measurements model some form of relative signal change, related to the contrast of interest, relative to the noise level. However, there is no consensus on how this contrast of interest should be conceptualised. Therefore, the scale of the CNR definitions varies widely and this makes comparing studies very hard.

### SNR and CNR definitions for fMRI data

**Figure 2:**
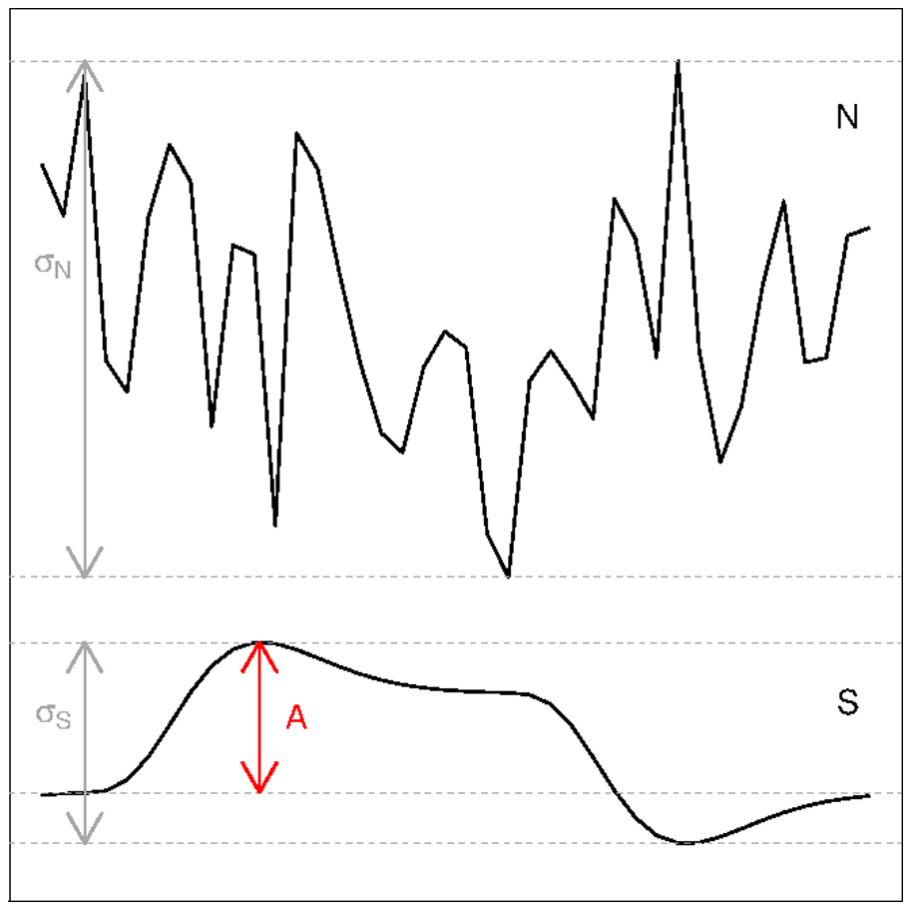
Illustration of the notation in the SNR and CNR definitions: 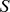 is the activation signal, 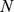 the noise signal, 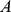 defines the amplitude of the activation signal, and 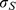 and 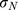 indicate the standard deviation of the activation signal and noise signal respectively.

Both SNR and CNR definitions have in common that a signal measure is compared to the noise level. The distinction between SNR versus CNR and the differences between the CNR definitions will be the result of how the signal measure and the noise is defined. While discussing the definitions, we will consider fMRI time series as the result of an addition of an activation signal time course and a noise signal time course. Figure 2 illustrates the notation we will use to define the signal and the noise. The activation signal time course, denoted as 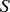, contains both the baseline signal and the possible fluctuations in the signal due to the experimental task. In general, 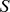 can be calculated as the average haemodynamic response function (HRF) of the fMRI time series in a certain ROI [18]. The noise signal, 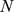, will typically be the composition of several noise sources such as system noise, physiological noise and task-related noise. When referring to the noise signal, we implicitly take into account all these sources, ignoring the specific influence or distribution of these sources [7]. To calculate 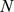 from the fMRI series in an ROI, the contribution of the activation signal can be reduced by subtracting the average HRF from the time series [18].

In the overview of the SNR and CNR definitions below, we will focus on those definitions that are commonly found in the literature. In fMRI simulation studies, values for SNR/CNR are often chosen to give an indication of the strength of the modelled signal relative to the modelled noise. Six different definitions were found in total. We will discuss their definition and whether they should be referred to as SNR or CNR. Note that, although in most papers these formulae were labelled as SNR, the majority of them are in fact CNR measurements.

#### Definition 1 (SNR)

The first definition models SNR based on the mean signal of the fMRI time series and the standard deviation of the noise in the time series [19], [20],
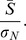



As such, the global signal level, comprised of the baseline and activation, is related to the noise.

#### Definition 2 (CNR)

In contrast, an amplitude measurement can be related to the standard deviation of the noise [21]–[24] and [25],
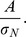



The amplitude of the signal is generally defined as the absolute difference between the baseline of the signal and the signal peak (Figure 2).

#### Definition 3 (CNR)

The previous definition of the CNR can also be transformed in decibel (dB) scale, which is a common scale in signal processing [26]–[28],
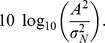



#### Definition 4 (CNR)

Another possibility is to model the strength of the signal based on the standard deviation of the activation signal [29]–[35],
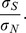



This definition is also implemented in the DCM simulator [36] and is a very intuitive measurement of CNR because the ratio of the fluctuations of both activation signal and noise is calculated.

#### Definition 5 (CNR)

[37] and [38] used the ratio of the variances,
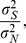
which is of course equal to the square of Definition 4.

#### Definition 6 (CNR)

Again, the ratio of the standard deviations is also found in dB scale [39]–[44],
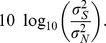



### Comments on the definitions

**Figure 3:**
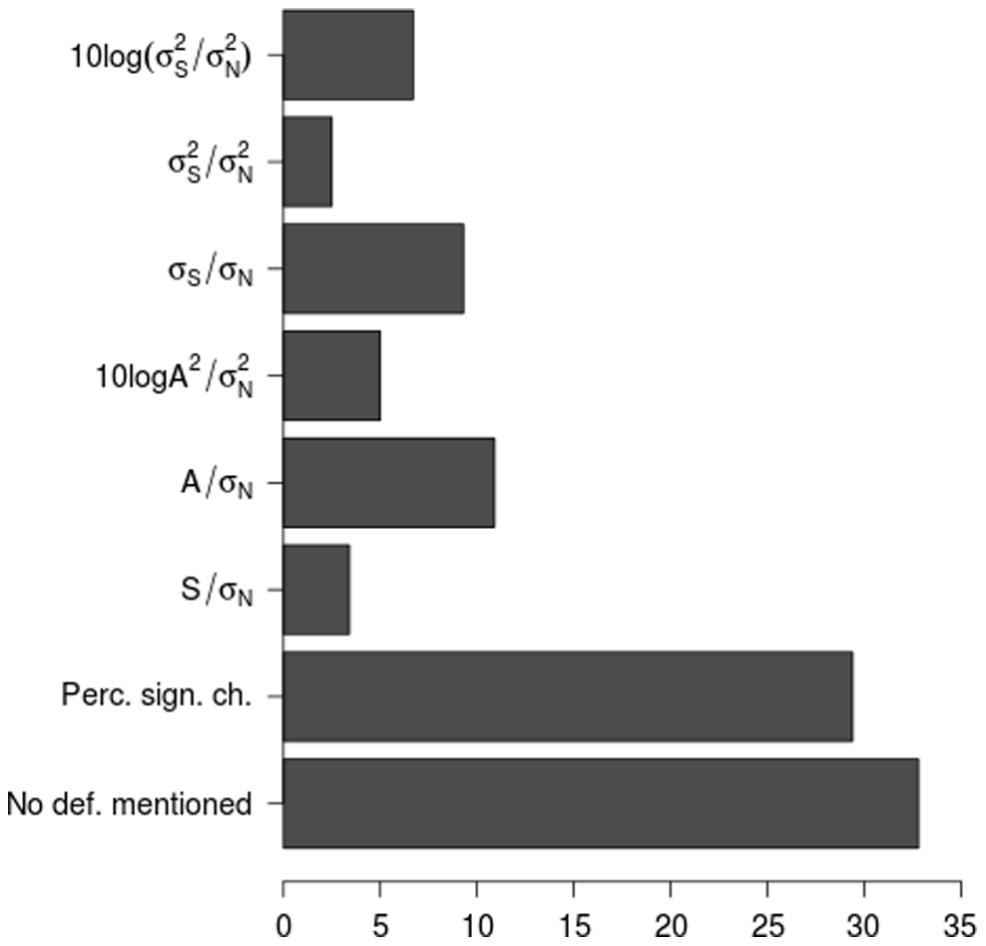
Percentage of the 119 simulation studies from the literature search that reported a specific SNR/CNR definition.

Figure 3 provides an overview of the frequencies in which the definitions discussed above are reported in fMRI simulation studies. About one third of the studies does not mention any SNR value, another third defines separate parameters for percent signal change (perc. sign. ch.) of the activation signal and for the noise level. The remainder of the studies mentions one of the definitions of which definitions 2 and 4 seem to be the most popular.

Definition 1 is actually a measurement of tSNR [9]. Baseline levels are highly dependent on the specific scanning parameters that are used to acquire the fMRI data. Moreover, because the BOLD signal fluctuations are very small, no real information about the activation signal strength is included in this definition, which makes it possibly not very suitable for task-related fMRI data. In fact, the higher the baseline value of the data, the less impact the activation signal will have on the value of the SNR. Therefore, the SNR value of a certain voxel in itself will not be informative to distinguish active from non-active voxels. On the other hand, tSNR can be very useful to evaluate resting-state fMRI, because it provides very accessible and easy to interpret information on the variation of the noise level over the brain.

In contrast, the remainder of the definitions all include some measurement of the activation signal strength. Therefore, these definitions are referred to as CNR formulae. It should be clear that, in theory, the value for these CNR definitions will always be zero for non-active voxels and larger than zero for active voxels. Consequently, theoretically it would be possible to detect active voxels based on their CNR value. In practice however, the activation signal is stricto sensu unknown and it may be complicated to calculate CNR values for single voxels.

For the CNR definitions, two different sets can be distinguished; the first set (Definition 2–3) focuses on the amplitude of the activation signal, 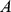, while the second set (Definition 4–6) incorporates the standard deviation of the activation as the signal of interest. With regard to the first set, these formulae can be interpreted as definitions of effect size based on means or differences between means, like for example Cohen's 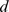 [45]. As such it is a direct indication of the strength of the signal.

Note that in our literature search, we also found possible negative SNR values, which might be confusing to many neuroscientists. These negative values stem from definitions 3 and 6 that define CNR on the dB scale, which are often used in signal processing. On this logarithmic scale, a value of 0 means that an equal amount of signal compared to noise is present in the data. Therefore, negative values are an indication of less signal than noise, while positive CNR values according to these definitions represent more activation signal than noise.

In the case of a block activation signal (Figure 2), the determination of the amplitude 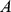 is quite straightforward. However, this is not the case in, for example, an event-related design. In this experimental design, it is typical that multiple events will cause several peaks in the signal and the timing of the stimuli will have an effect on the height of the peak. In this case, the amplitude of the signal could be either the difference between the baseline and the maximal height of the signal, or the mean amplitude over all peaks. In contrast, calculating the standard deviation of the activation signal, 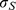, is independent of the experimental design (i.e. block or event-related designs).

So far, the definitions described above were only discussed based on a single condition experiment. As soon as multiple conditions are considered in a experiment, it is not quite clear anymore how to calculate the SNR or CNR of the fMRI data. One option could be to determine the SNR/CNR for each condition separately, which would be valid when distinct regions are activated by the conditions. Another option could be to first create an expected activation signal based on a contrast between the conditions, and then to calculate the SNR/CNR of the contrast signal in the same manner as for single condition time series. In this way, the signal of interest is directly based on the contrast that will be tested.

In essence all of these definitions have the same denominator (i.e. 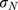) so that differences are just scaling differences based on the definition of the activation signal. One desirable property for an SNR or CNR definition of fMRI time series would be that it is closely related to the activation detection power. If the SNR/CNR is high, then the power should be high too (keeping everything else constant). Secondly, the scaling differences make it hard to compare the values of the discussed definitions. In the remainder of this paper, we will present some tools that will enable comparison among the different definitions and further, we will shed some light on the relationship with activation detection power.

### Comparing the SNR and CNR values

Due to the fact that there is no consensus on how to define the SNR or CNR for fMRI data, interpreting a value can be an almost impossible job. Dependent on how the SNR/CNR is calculated, the values will be on a different scale. This impedes comparability between fMRI studies and consequently delays convergence of conclusions. In order to facilitate the comprehension of SNR and CNR values, three reference tables were assembled (Table 1–3), based on the three experimental designs that were also used in the simulation study. For all levels of the noise and activation parameters, the SNR or CNR according to the six definitions was calculated and the results are presented in Table 1, Table 2 and Table 3. Note that for the ER design the amplitude was defined as the maximal amplitude (i.e. amplitude of the highest peak). In the case of the contrast design, the SNR and CNR values were calculated based on the contrast signal that was the difference of the activation signals of the two conditions. The amplitude of this contrast signal was calculated as the difference between the maximum and the minimum.

**Table 1:** Reference table for the different SNR/CNR definitions based on a block design.

% Sig. ch.	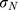	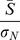	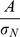	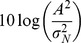	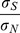	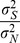	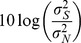	Power
1	0.1	1003	10	20	4.46	19.85	12.98	1.00
	0.2	502	5	14	2.23	4.96	6.96	1.00
	0.5	201	2	6	0.89	0.79	−1.00	1.00
	1	100	1	0	0.45	0.20	−7.02	0.99
	2	50	0.5	−6	0.22	0.050	−13.04	0.58
	5	20	0.2	−14	0.089	0.0079	−21.00	0.14
	10	10	0.1	−20	0.045	0.0020	−27.02	0.07
2	0.1	1007	20	26	8.91	79.42	19.00	1.00
	0.2	503	10	20	4.46	19.85	12.97	1.00
	0.5	201	4	12	1.78	3.18	5.02	1.00
	1	101	2	6	0.89	0.79	−1.00	1.00
	2	50	1	0	0.45	0.20	−7.02	0.99
	5	20	0.4	−8	0.18	0.032	−14.98	0.42
	10	10	0.2	−14	0.089	0.0079	−21.00	0.15
5	0.1	1017	50	34	22.28	496.35	26.96	1.00
	0.2	508	25	28	11.14	124.09	20.94	1.00
	0.5	203	10	20	4.46	19.85	12.98	1.00
	1	102	5	14	2.23	4.96	6.96	1.00
	2	51	2.5	8	1.11	1.24	0.94	1.00
	5	20	1	0	0.45	0.1985	−7.02	0.99
	10	10	0.5	−6	0.22	0.0496	−13.04	0.59

**Table 2:** Reference table for the different SNR/CNR definitions based on an ER design.

% Sig. ch.	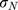	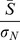	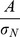	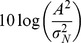	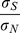	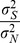	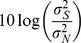	Power
1	0.1	1002	10	20	3.07	9.41	9.74	1.00
	0.2	501	5	14	1.53	2.35	3.72	1.00
	0.5	200	2	6	0.61	0.38	−4.24	0.99
	1	100	1	0	0.31	0.094	−10.26	0.67
	2	50	0.5	−6	0.15	0.024	−16.28	0.22
	5	20	0.2	−14	0.06	0.0038	−24.24	0.08
	10	10	0.1	−20	0.03	0.00094	−30.26	0.06
2	0.1	1004	20	26	6.14	37.64	15.76	1.00
	0.2	502	10	20	3.07	9.41	9.74	1.00
	0.5	201	4	12	1.23	1.51	1.78	1.00
	1	100	2	6	0.61	0.38	−4.24	0.99
	2	50	1	0	0.31	0.094	−10.26	0.75
	5	20	0.4	−8	0.13	0.015	−18.23	0.17
	10	10	0.2	−14	0.06	0.0038	−24.24	0.08
5	0.1	1010	50	34	15.34	235.26	23.72	1.00
	0.2	505	25	28	7.67	58.81	17.69	1.00
	0.5	202	10	20	3.07	9.41	9.74	1.00
	1	101	5	14	1.54	2.35	3.72	1.00
	2	51	2.5	8	0.77	0.59	−2.31	0.99
	5	20	1	0	0.31	0.15	−10.26	0.64
	10	10	0.5	−6	0.15	0.024	−16.28	0.21

**Table 3:** Reference table for the different SNR/CNR definitions based on a contrast.

% Sig. ch.	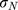	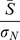	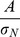	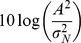	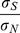	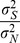	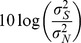	Power
1	0.1	1001	10.56	20.47	3.02	9.14	9.61	1.00
	0.2	501	5.28	14.45	1.51	2.28	3.59	1.00
	0.5	200	2.11	6.49	0.60	0.37	−4.37	0.96
	1	100	1.06	0.47	0.30	0.09	−10.39	0.46
	2	50	0.53	−5.55	0.15	0.02	−16.41	0.15
	5	20	0.21	−13.51	0.06	0.0037	−24.37	0.07
	10	10	0.11	−19.53	0.03	0.0009	−30.39	0.05
2	0.1	1003	21.12	26.49	6.05	36.56	15.63	1.00
	0.2	501	10.56	20.47	3.02	9.14	9.61	1.00
	0.5	201	4.22	12.51	1.21	1.46	1.65	1.00
	1	100	2.11	6.49	0.60	0.37	−4.37	1.00
	2	50	1.06	0.47	0.30	0.091	−10.39	0.92
	5	20	0.42	−7.49	0.12	0.015	−18.35	0.27
	10	10	0.21	−13.51	0.06	0.004	−24.37	0.10
5	0.1	1007	52.79	34.45	15.12	228.50	23.59	1.00
	0.2	504	26.40	28.43	7.56	57.12	17.57	1.00
	0.5	201	10.56	20.47	3.02	9.14	9.61	1.00
	1	101	5.28	14.45	1.51	2.28	3.59	1.00
	2	50	2.64	8.43	0.76	0.57	−2.43	0.99
	5	20	1.06	0.47	0.30	0.09	−10.39	0.47
	10	10	0.53	−5.55	0.15	0.02	−16.41	0.16

The results in Tables 1–3 demonstrate that the SNR definition (Definition 1) is highly dependent on the value of the baseline, since the formula is based on the mean signal strength. Additionally, the obtained values are almost invariant to changes in the activation signal strength and the experimental design.

The CNR definitions based on the amplitude of the signal (Definition 2 and Definition 3) are also partially determined by the baseline since the (maximal) amplitude of the signal will always correspond to the % signal change relative to the baseline. However, given the relative % signal change of the activation or contrast signal, the amplitude is constant over experimental designs. This is not true for the CNR definitions based on the standard deviation of the activation signal (Definition 4, Definition 5 and Definition 6). Although these CNR definitions are completely independent from the baseline, the activation standard deviation will be influenced by the number of events in an ER design or by the length of the epochs in a block design. The reference tables (Table 1, 2 and 3) illustrate this variation, but the close range of these CNR values over the designs indicates that this variation is rather small. Therefore, the reference tables presented here provide a tool to roughly compare and interpret the values for the different SNR/CNR definitions.

### Analytic similarities

Of course, the conversion of one definition to another can also be solved analytically in some cases. For completeness, we explicitly demonstrate here the analytic similarities between the SNR/CNR definitions. Given the percent signal change 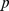 of the activation signal, the amplitude of the signal will be defined as
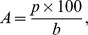
with 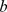 the baseline of the activation signal. A CNR value 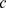 calculated based on Definition 2 or Definition 4 can be converted to a CNR value in dB, 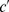 using



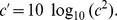



Vice versa, a dB CNR value 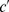 can be back transformed to the CNR in the original scale, 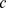, by
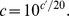



Since the standard deviation of the activation signal (as in Definition 4–6) will be partially determined by the experimental design, there is no direct way to go from the percent signal change to the standard deviation. To compare these CNR values, either the reference tables, listed here, can be used to provide a rough estimate, or the values have to be calculated specifically for each design.

### The relationship with detection power

There is no discussion on the fact that SNR or CNR is somehow related to activation detection power. Indeed, the higher the signal or the lower the noise (i.e. higher values for the SNR/CNR), the higher the power will be. Naively, one could expect that, when, for example, SNR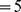 and the power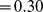, the power will increase to 0.60 for data with an SNR of 10. In other words, one may expect an approximate linear relationship between SNR/CNR values and the power to detect activation. In order to establish the approximate relationship between activation detection power and the SNR/CNR definitions, power results are presented in the last column of the reference tables (Tables 1–3). Note that these results represent maximal power values. In real fMRI data, the power will be smaller due to the influence of non-white noise.

**Figure 4:**
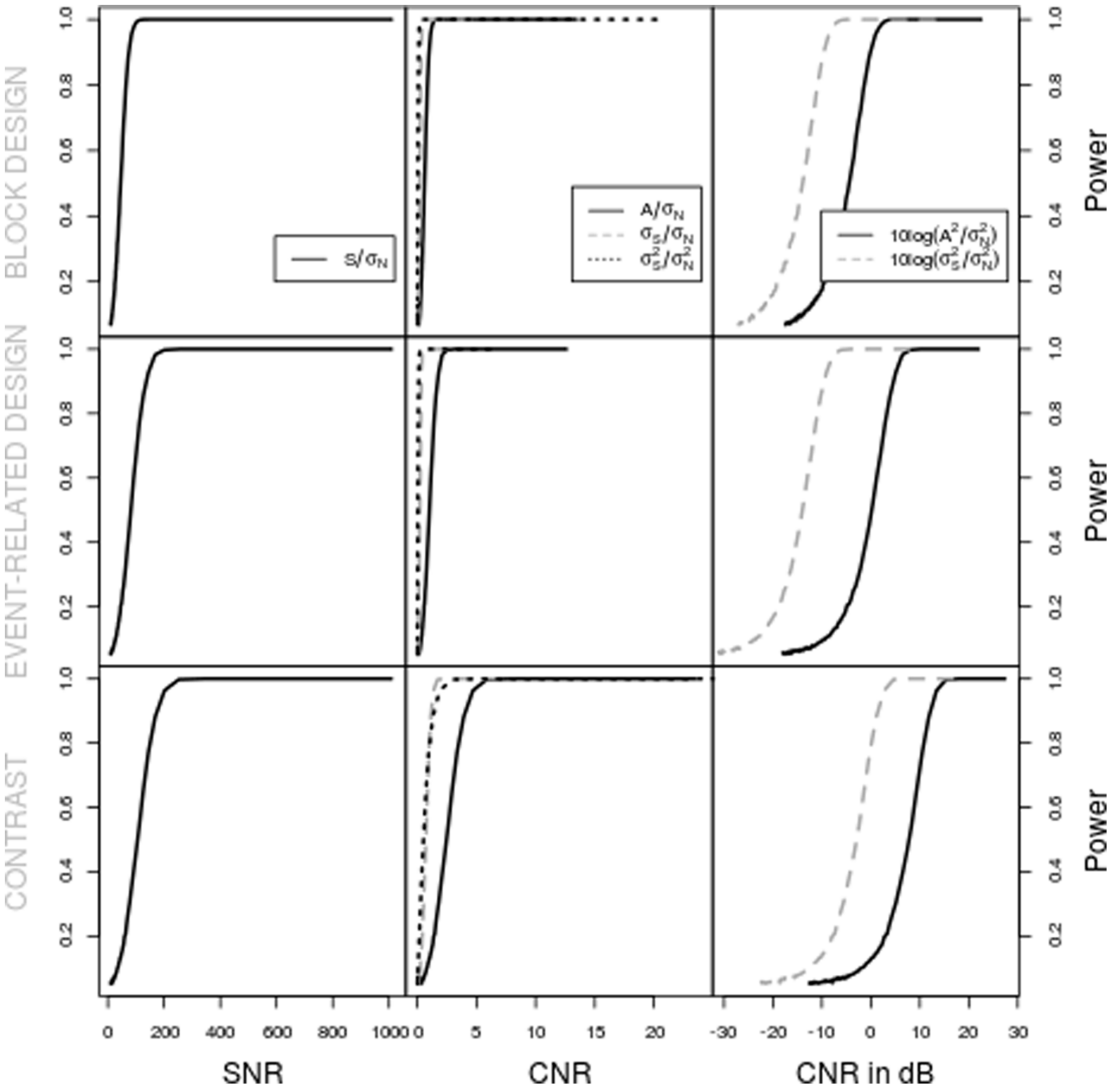
The relationship between power and the SNR/CNR definitions for 1% signal change.

Looking at the results, we can immediately conclude that the simple rule *“twice as much signal will double the power”* is not valid. Indeed, as power is bounded, a linear relationship with the signal is impossible. In general, the power will be lower for the time series that contain more noise, but their is no linear relationship with the SNR or CNR values. This is illustrated in Figure 4 for the case of 1% signal change. However, comparing the power values for the different designs, overall lower values can be observed for the ER design notwithstanding equal activation strengths and noise levels. This lower power is in itself not that surprising, but this can only be predicted based on the CNR definitions that use the standard deviation of the activation signal, since the SNR/CNR values for the other definitions are constant over the designs. Additionally, in the lower power cases, the CNR values of Definition 4 are within the same range, indicating that these CNR values can be used as a rough estimate of activation detection power.

### Real data example

**Figure 5:**
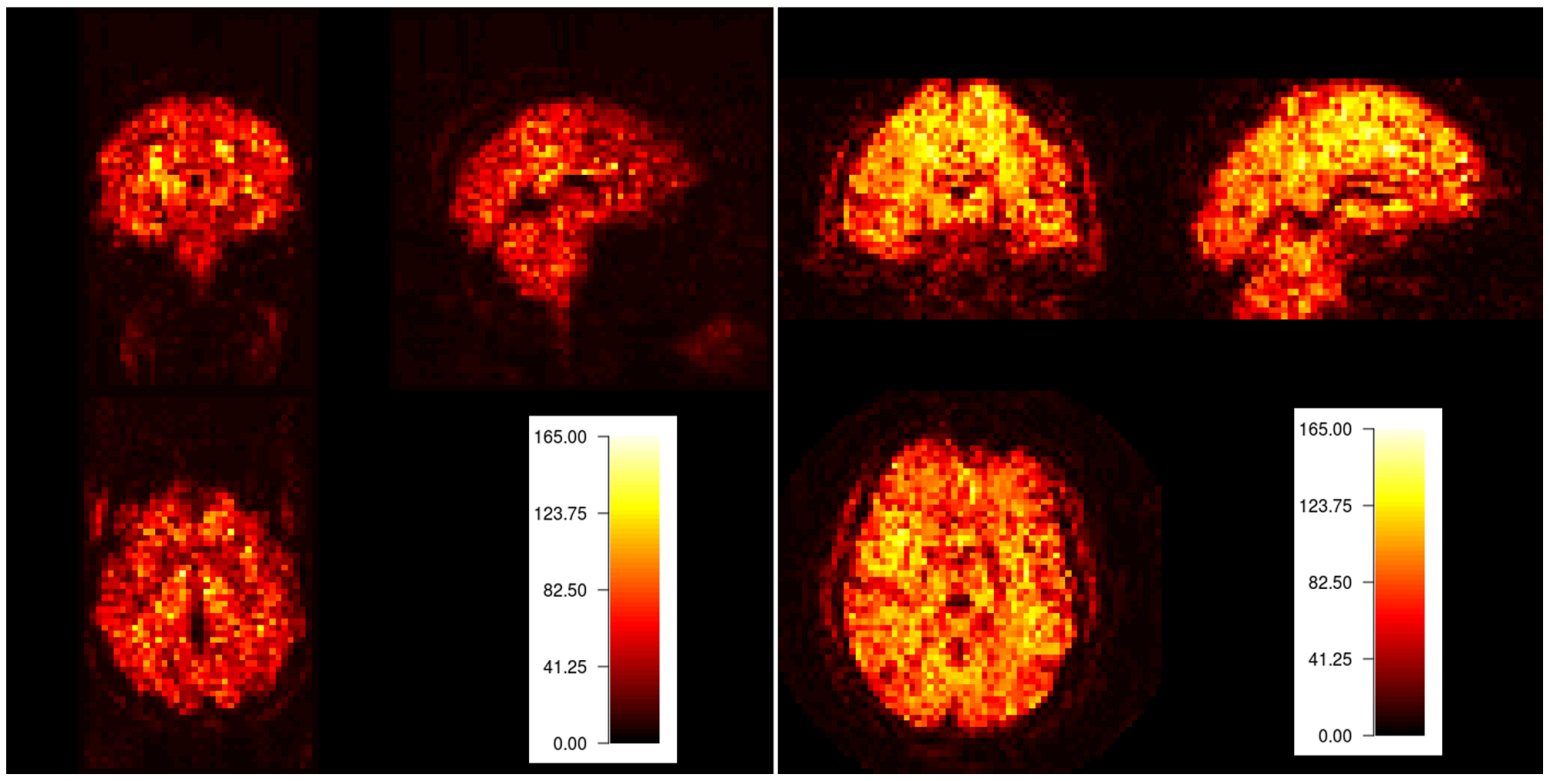
tSNR results of the example data, illustrating how the definitions can be applied to real task-based and resting-state fMRI data. Upper panel: tSNR results for a block design. Lower panel: tSNR results for the resting-state data.

For both datasets we calculated the tSNR (Definition 1), the amplitude-based CNR (Definition 2) and the standard deviation CNR (Definition 4) on the raw data. The tSNR was calculated similarly for both datasets. For each voxel the mean and the standard deviation of the corresponding time series were calculated and then divided to determine the tSNR. Note that the interpretation of tSNR is only useful in gray matter. The results are presented in Figure 5 (upper panel). The tSNR of the task-based data is on average 10.83 and ranges between 0.03 and 161.20. For the resting-state data, the mean tSNR is 12.98, ranging from 1.07 to 84.54. Based on these results, it seems that the data quality of the resting-state data is higher compared to the task-based data. Figure 5 (upper panel) also shows that the spatial distribution of the tSNR values is more equal for the resting-state data (right) than for the task-based data (left).

CNR values are only informative for the task fMRI data, so they were not determined for the resting state data. To calculate them, we first had to create an activation signal. For the task-based data, the activation signal was calculated by averaging the time series of all voxels within the activation contrast mask (i.e. the mask indicating the mean response over all categories). A second activation signal was calculated for all voxels outside the mask. These activation signals were then subtracted from the individual voxels time series to eliminate the contribution of activation to the time series and isolate the noise. The resulting signals, both activation signals and the noise signal, were used to calculate the parameters for the CNR definitions.

The amplitude-based CNR measure (left) is on average 37.06 (with a range from 8.17 to 95.73) for in-mask voxels and 0.48 (with a range from 0.01 to 95.73) for out-mask voxels. Similarly, for the CNR measure using the standard deviation of the signal, in-mask voxels had an average CNR of 0.029 (ranging from 0.06 to 0.76), while out-mask voxels had an average CNR of 0.04 (ranging from 0.01–0.76). Of course, it should be noted that these values are highly dependent on how the mask or ROIs are determined.

## Discussion

fMRI data are often characterised by their SNR or CNR. SNR measurements are, for example, used to compare scanner hardware or the quality of scanning sequences, while CNR can be indicative of the quality (i.e. detectability) of the contrast of interest. In this paper, an overview was provided of common SNR and CNR definitions in an fMRI time series context. It was established that the literature lacks consensus on how to define SNR/CNR for fMRI data. Consequently, reported SNR and CNR values are hard to compare, possibly hindering the convergence of conclusions based on fMRI studies.

Based on how the signal of interest is defined, an explicit distinction was made between SNR and CNR. SNR compares the global signal level to the amount of noise and can be applied to either MRI images or task-related and resting-state fMRI (e.g. tSNR). The main purpose of determining the SNR of the data will be to assess the quality of the data (e.g. influence of noise). However, when applied to task-related fMRI data, the SNR of the data will most likely miss out on the small fluctuations present in the activation signal that are caused by the task. Therefore, in the case of these particular data, in which the signal of interest is a specific contrast that models the influence of certain conditions, it would be better to consistently use the concept of CNR. The CNR value will also give an indication of the quality of the data in terms of noise, but additionally it contains information on the strength of the activation signal for a specific task. This information can be related to activation detection sensitivity.

A sceptical reader would argue that it might be meaningless to capture the information present in 4D fMRI data, which are characterised by very high inter- and intra-subject and -scanner variability, in one single number (either SNR or CNR). Indeed, for real data, SNR or CNR values are seldom reported. Moreover, screening of published simulation studies teaches us that no less than 62.2% of these studies avoid reporting an SNR/CNR value. Instead, they reported separate parameters for the activation strength and the noise level. A second problem might be that the same value of SNR/CNR can indicate different levels of activation strength and noise, which can have a different impact on the detection accuracy. Despite the justly scepticism, determining the SNR or CNR of fMRI data can still hold useful information, because it provides an assesment of the quality of the data at a glance. However, we recommend to calculate the values only for small regions that are likely to have the same value of SNR/CNR based on anatomy or function. For simulation studies in particular, it would be interesting to report the SNR/CNR of the simulated value along with the specific values of activation strength and noise level. As such, generalising the conclusions from these studies to real data will be facilitated.

## Conclusion

Consensus on a common SNR/CNR definition for fMRI data might be difficult to achieve, because the measurement depends very much on how the signal of interest and the noise is defined. Therefore, we strongly recommend that authors reporting SNR/CNR values, at least mention the type of definition they use and provide an interpretation on the meaning of the reported values. The tables presented in this chapter can then be a reference allowing easy comparison from one definition to another. Furthermore, these tables are an excellent tool to provide an estimate of the maximal power that can be expected to detect activation in data with a given SNR/CNR value. Finally, a better understanding of the SNR/CNR values might encourage fMRI researchers to report these measurements in a more systematic way. Consequently, the ability to compare these reported values will facilitate the convergence of fMRI based knowledge.
